# 1-(2-Fluoro­phen­yl)-6,7-dimethoxy­isochroman

**DOI:** 10.1107/S160053680900926X

**Published:** 2009-03-25

**Authors:** Aamer Saeed, Jim Simpson, Roderick G. Stanley

**Affiliations:** a Department of Chemistry, Quaid-i-Azam University, Islamabad 45320, Pakistan; b Department of Chemistry, University of Otago, PO Box 56, Dunedin, New Zealand

## Abstract

In the title compound, C_17_H_17_FO_3_, the benzene ring of the isochroman unit is inclined at 84.96 (7)° to the fluoro­benzene ring plane, and the pyran ring adopts a half-boat conformation. In the crystal structure, C—H⋯O hydrogen bonds link mol­ecules into rows along the *c* axis, while C—H⋯O inter­actions and C—H⋯F hydrogen bonds to the fluorine acceptor stack the mol­ecules down the *b* axis. In addition, the crystal structure exhibits a weak C—H⋯π inter­action between a methyl H atom of the meth­oxy group and the dimethoxy­benzene ring of an adjacent mol­ecule.

## Related literature

For details of naturally occurring isochromans, see: Imamura *et al.* (2000 ▶); Ogawa *et al.* (2004 ▶); Peng *et al.* (1999 ▶); Kunesch *et al.* (1987 ▶). For the biological activity of isochromans, see: Zhang *et al.* (2008 ▶); Lorenz *et al.* (2005 ▶); Togna *et al.* (2003 ▶); Bianchi *et al.* (2004 ▶); Cutler *et al.* (1997 ▶); Liu *et al.* (2005 ▶); TenBrink *et al.* (1996 ▶); Frater *et al.* (1999 ▶); Dobson & Humber (1975 ▶); Yamato *et al.* (1985 ▶); McCall *et al.* (1982 ▶). For the synthesis of isochromans, see: Guiso *et al.* (2001 ▶). For related structures, see: Saeed & Flörke (2006*a* ▶,*b* ▶). For ring puckering analysis, see: Cremer & Pople (1975 ▶); and for reference structural data, see: Allen *et al.* (1987 ▶).
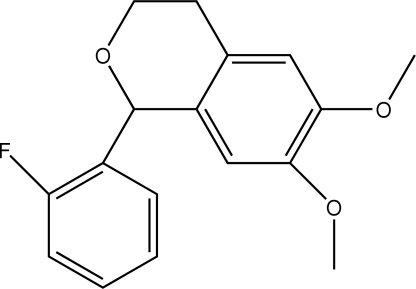


## Experimental

### 

#### Crystal data

C_17_H_17_FO_3_
*M* *_r_* = 288.31
Monoclinic, 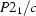

*a* = 15.730 (2) Å
*b* = 5.2328 (8) Å
*c* = 16.477 (2) Å
β = 93.108 (8)°
*V* = 1354.3 (3) Å^3^
*Z* = 4
Mo *K*α radiation
μ = 0.11 mm^−1^
*T* = 89 K
0.29 × 0.22 × 0.13 mm

#### Data collection

Bruker APEXII CCD area-detector diffractometer
Absorption correction: multi-scan (*SADABS*; Bruker, 2006 ▶) *T*_min_ = 0.789, *T*_max_ = 0.986
13466 measured reflections
2371 independent reflections
1864 reflections with *I* > 2σ(*I*)
*R*_int_ = 0.072

#### Refinement

*R*[*F* ^2^ > 2σ(*F* ^2^)] = 0.074
*wR*(*F* ^2^) = 0.261
*S* = 1.28
2371 reflections
193 parameters
H-atom parameters constrained
Δρ_max_ = 0.45 e Å^−3^
Δρ_min_ = −0.37 e Å^−3^

### 

Data collection: *APEX2* (Bruker, 2006 ▶); cell refinement: *APEX2* and *SAINT* (Bruker, 2006 ▶); data reduction: *SAINT*; program(s) used to solve structure: *SHELXS97* (Sheldrick, 2008 ▶); program(s) used to refine structure: *SHELXL97* (Sheldrick, 2008 ▶) and *TITAN2000* (Hunter & Simpson, 1999 ▶); molecular graphics: *SHELXTL* (Sheldrick, 2008 ▶) and *Mercury* (Macrae *et al.*, 2006 ▶); software used to prepare material for publication: *SHELXL97*, *enCIFer* (Allen *et al.*, 2004 ▶), *PLATON* (Spek, 2009 ▶) and *publCIF* (Westrip, 2009 ▶).

## Supplementary Material

Crystal structure: contains datablocks global, I. DOI: 10.1107/S160053680900926X/lx2092sup1.cif

Structure factors: contains datablocks I. DOI: 10.1107/S160053680900926X/lx2092Isup2.hkl

Additional supplementary materials:  crystallographic information; 3D view; checkCIF report

## Figures and Tables

**Table 1:** Hydrogen-bond geometry (Å, °)

*D*—H⋯*A*	*D*—H	H⋯*A*	*D*⋯*A*	*D*—H⋯*A*
C1—H1*B*⋯O2^i^	0.99	2.59	3.360 (5)	134
C7—H7⋯F1^ii^	0.95	2.45	3.360 (4)	160
C17—H17*B*⋯O1^iii^	0.98	2.49	3.430 (4)	160
C17—H17*A*⋯*Cg*^ii^	0.98	2.70	3.557 (3)	146

Symmetry codes: (i) 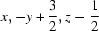; (ii) 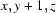; (iii) 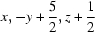. *Cg*2 is the centroid of the C3–C8 benzene ring.

**Fig. 1.**
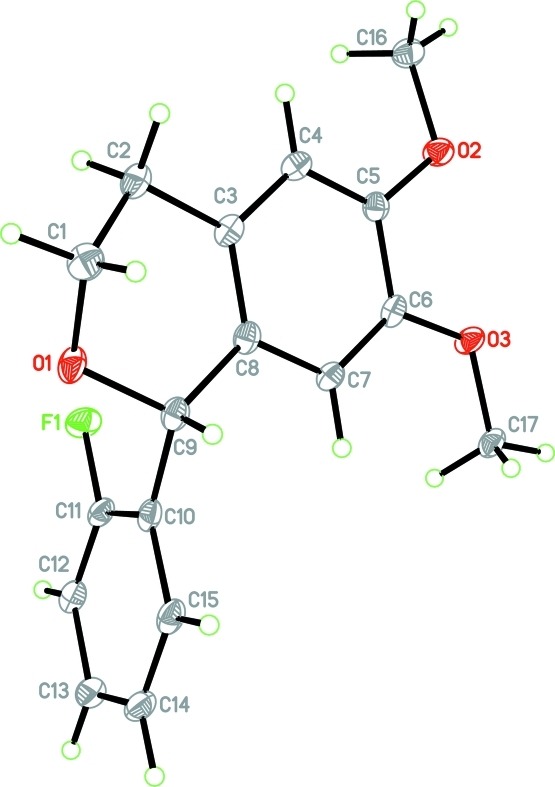
The molecular structure of the title compound, showing displacement ellipsoids drawn at the 50% probability level.

**Fig. 2.**
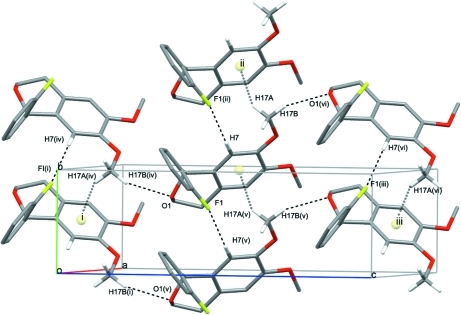
C—H···F, C—H···O (dashed lines) and C—H···π interactions (dotted lines) in the title compound. The yellow spheres denote the ring centroids [symmetry codes: (i) x, 1.5-y,-1/2+z; (ii) x, 1+y,z; (iii) x, 1.5-y, 1/2+z; (iv) x, 2.5-y, -1/2+z; (v) x, -1+y, z; (vi) x, 2.5-y, 1/2+z ].

**Fig. 3.**
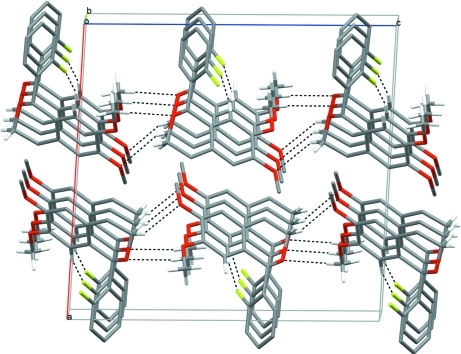
Crystal packing for (I) viewed down the b axis with hydrogen bonds drawn as dashed lines and H atoms on atoms not involved in hydrogen bonding omitted.

## supplementary crystallographic information

### Comment

Isochroman is a common structural motif found in many natural products. For example 1,6,8-trihydroxy-3-heptyl-7-carboxyisochroman, is an antibiotic and topoisomerase II inhibitor from Penicillum *sp*. (Imamura *et al.*, 2000), pseudodeflectusin is a selective human cancer cytotoxin from Aspergillus pseudodeflectus, (Ogawa *et al.*, 2004), in softwood lignin (Peng *et al.*, 1999) and in the male wing gland pheromone of Aphomia sociella (Kunesch *et al.*, 1987). A novel isochroman derivative inhibited apoptosis in vascular endothelial cells by depressing the levels of integrin 4, p53 and ROS (Zhang *et al.*, 2008). 1-Phenyl- and 1-(3-methoxy-4-hydroxy)phenyl-6,7-dihydroxyisochromans identified in extra-virgin olive oil exhibit beneficial antioxidant effects (Lorenz *et al.*, 2005) and antiplatelet activity (Togna *et al.*, 2003). Isochroman derivatives also show plant-growth regulatory and herbicidal activities (Bianchi *et al.*, 2004; Cutler *et al.*, 1997), these are oestrogen receptors (Liu *et al.*, 2005), dopamine receptor ligands (TenBrink *et al.*, 1996), and fragrances, such as galaxolide (Frater *et al.*, 1999). 1-Aryl-6,7-dimethoxyisochromans are known to demonstrate analgesic, muscle relaxant, antidepressant, antiinflammatory, antihistaminic and anticoagulant activity and are adrenergic antagonists (Dobson & Humber 1975; Yamato *et al.*, 1985; McCall *et al.*, 1982). The title dimethoxyisochroman derivative (I), Fig. 1, was prepared by the oxa-Pictet–Spengler reaction for the preparation of isochromans (Guiso *et al.*, 2001) using 2-(3,4-dimethoxyphenyl)ethanol and 2-fluorobenzaldehyde.

The pyran ring of (I) adopts a half-boat conformation (Cremer & Pople, 1975) with the O1 atom 0.639 (3) Å from the least-squares plane through atoms C1–C3, C8, C9.  The r.m.s. deviation from this plane was 0.083 Å. The benzene ring of the isochroman unit is inclined at 84.96 (7) ° to the fluorobenzene ring plane. Both the C and O atoms of the two methoxy substituents lie close to the aromatic ring plane (maximum deviation 0.310 (5) Å for C16).

In the molecular packing (Fig. 2),  C17—H17B···O1 hydrogen bonds link the molecules into rows along the c axis (Fig. 2 and Table 1; symmetry codes as in Fig. 2). The F1 atom acts as an acceptor in a C7—H7···F1 hydrogen bond that, together with C1—H1B···O2 interactions, stacks molecules from individual rows down the the b axis (Fig. 2, Fig 3 and Table 1; symmetry codes as in Fig. 2). Additionally, a weak C—H···π interaction in the structure was observed between a methyl H atom of the methoxy group and the dimethoxybenzene ring of an adjacent molecule, with a C17—H17A···Cg^i^ separation of 2.70 Å (Table 1 and Fig. 2; Cg is the centroid of the C3–C8 benzene ring, symmetry codes as in Fig. 2).)

### Experimental

A homogenized mixture of 2-(3,4-dimethoxyphenyl)ethanol (0.18g, 1 mmol) and 4-fluorobenzaldehyde (0.12g 1 mmol) and a catalytic amount of *p*-toluenesulfonic acid monohydrate was irradiated for 1.3 min. The product was purified by thin layer chromatography using petroleum ether and ethyl acetate (7:2 v:v) to afford the title compound (0.91 mmol, 91%) which was recrystallized from ethyl acetate. Analysis calculated for C_17_H_17_O_3_F: C, 70.82%, H, 5.94% found, 70.69%, H, 5.97%.

### Refinement

All H-atoms were positioned geometrically and refined using a riding model with d(C—H) = 0.95 Å, *U*_iso_=1.2*U*_eq_ (C) for aromatic 1.00 Å, *U*_iso_ = 1.2*U*_eq_ (C) for CH, 0.99 Å, *U*_iso_ = 1.2*U*_eq_ (C) for CH_2_ and 0.98 Å, *U*_iso_ = 1.5*U*_eq_ (C) for CH_3_ hydrogen atoms.

### Crystal data

**Table:**

C_17_H_17_FO_3_	*F*(000) = 608
*M**_r_* = 288.31	*D*_x_ = 1.414 Mg m^−^^3^
Monoclinic, *P*2_1_/*c*	Mo *K*α radiation, λ = 0.71073 Å
Hall symbol: -P 2ybc	Cell parameters from 3061 reflections
*a* = 15.730 (2) Å	θ = 2.5–28.7°
*b* = 5.2328 (8) Å	µ = 0.11 mm^−^^1^
*c* = 16.477 (2) Å	*T* = 89 K
β = 93.108 (8)°	Irregular fragment, colourless
*V* = 1354.3 (3) Å^3^	0.29 × 0.22 × 0.13 mm
*Z* = 4	

### Data collection

**Table:**

Bruker APEXII CCD area-detector diffractometer	2371 independent reflections
Radiation source: fine-focus sealed tube	1864 reflections with *I* > 2σ(*I*)
graphite	*R*_int_ = 0.072
Detector resolution: 10.0 pixels mm^-1^	θ_max_ = 25.0°, θ_min_ = 2.6°
ω' scans	*h* = −17→18
Absorption correction: multi-scan (*SADABS*; Bruker, 2006)	*k* = −6→5
*T*_min_ = 0.789, *T*_max_ = 0.986	*l* = −19→19
13466 measured reflections	

### Refinement

**Table:**

Refinement on *F*^2^	Secondary atom site location: difference Fourier map
Least-squares matrix: full	Hydrogen site location: inferred from neighbouring sites
*R*[*F*^2^ > 2σ(*F*^2^)] = 0.074	H-atom parameters constrained
*wR*(*F*^2^) = 0.261	*w* = 1/[σ^2^(*F*_o_^2^) + (0.1483*P*)^2^ + 0.6898*P*] where *P* = (*F*_o_^2^ + 2*F*_c_^2^)/3
*S* = 1.28	(Δ/σ)_max_ < 0.001
2371 reflections	Δρ_max_ = 0.45 e Å^−^^3^
193 parameters	Δρ_min_ = −0.37 e Å^−^^3^
0 restraints	Extinction correction: *SHELXS97* (Sheldrick, 2008), Fc^*^=kFc[1+0.001xFc^2^λ^3^/sin(2θ)]^-1/4^
Primary atom site location: structure-invariant direct methods	Extinction coefficient: 0.021 (8)

### Special details

**Table:**

Geometry. All esds (except the esd in the dihedral angle between two l.s. planes) are estimated using the full covariance matrix. The cell esds are taken into account individually in the estimation of esds in distances, angles and torsion angles; correlations between esds in cell parameters are only used when they are defined by crystal symmetry. An approximate (isotropic) treatment of cell esds is used for estimating esds involving l.s. planes.
Refinement. Refinement of F^2^ against ALL reflections. The weighted R-factor wR and goodness of fit S are based on F^2^, conventional R-factors R are based on F, with F set to zero for negative F^2^. The threshold expression of F^2^ > 2sigma(F^2^) is used only for calculating R-factors(gt) etc. and is not relevant to the choice of reflections for refinement. R-factors based on F^2^ are statistically about twice as large as those based on F, and R- factors based on ALL data will be even larger.

### Fractional atomic coordinates and isotropic or equivalent isotropic displacement parameters (Å^2^)

**Table:**

	*x*	*y*	*z*	*U*_iso_*/*U*_eq_	
O1	0.23025 (15)	0.7414 (5)	0.31621 (15)	0.0181 (7)	
C1	0.3182 (2)	0.7001 (7)	0.3030 (2)	0.0203 (9)	
H1A	0.3425	0.8551	0.2787	0.024*	
H1B	0.3240	0.5568	0.2644	0.024*	
C2	0.3667 (2)	0.6388 (7)	0.3826 (2)	0.0177 (9)	
H2A	0.3524	0.4635	0.3999	0.021*	
H2B	0.4287	0.6455	0.3749	0.021*	
C3	0.3445 (2)	0.8270 (7)	0.4478 (2)	0.0151 (8)	
C4	0.3972 (2)	0.8448 (6)	0.5191 (2)	0.0149 (8)	
H4	0.4458	0.7373	0.5257	0.018*	
C5	0.3797 (2)	1.0161 (6)	0.5799 (2)	0.0140 (8)	
O2	0.42783 (15)	1.0436 (5)	0.65158 (15)	0.0173 (7)	
C16	0.4888 (2)	0.8446 (7)	0.6690 (2)	0.0199 (9)	
H16A	0.4608	0.6779	0.6626	0.030*	
H16B	0.5124	0.8627	0.7250	0.030*	
H16C	0.5348	0.8571	0.6314	0.030*	
C6	0.3085 (2)	1.1783 (6)	0.5699 (2)	0.0143 (8)	
O3	0.29623 (15)	1.3416 (5)	0.63316 (15)	0.0167 (7)	
C17	0.2271 (2)	1.5183 (7)	0.6224 (2)	0.0167 (8)	
H17A	0.2353	1.6245	0.5745	0.025*	
H17B	0.2251	1.6275	0.6706	0.025*	
H17C	0.1734	1.4238	0.6148	0.025*	
C7	0.2562 (2)	1.1589 (6)	0.4993 (2)	0.0143 (8)	
H7	0.2078	1.2665	0.4922	0.017*	
C8	0.2740 (2)	0.9841 (6)	0.4389 (2)	0.0143 (8)	
C9	0.2186 (2)	0.9759 (7)	0.3601 (2)	0.0154 (8)	
H9	0.2368	1.1196	0.3250	0.019*	
C10	0.1238 (2)	1.0018 (6)	0.3687 (2)	0.0146 (8)	
C11	0.0776 (2)	0.8291 (6)	0.4129 (2)	0.0145 (8)	
F1	0.12090 (13)	0.6419 (4)	0.45485 (12)	0.0202 (6)	
C12	−0.0089 (2)	0.8363 (6)	0.4168 (2)	0.0164 (8)	
H12	−0.0378	0.7140	0.4479	0.020*	
C13	−0.0540 (2)	1.0280 (7)	0.3740 (2)	0.0185 (9)	
H13	−0.1142	1.0358	0.3750	0.022*	
C14	−0.0104 (2)	1.2076 (7)	0.3300 (2)	0.0193 (9)	
H14	−0.0409	1.3394	0.3014	0.023*	
C15	0.0770 (2)	1.1947 (7)	0.3276 (2)	0.0162 (8)	
H15	0.1061	1.3190	0.2975	0.019*	

### Atomic displacement parameters (Å^2^)

**Table:**

	*U*^11^	*U*^22^	*U*^33^	*U*^12^	*U*^13^	*U*^23^
O1	0.0232 (14)	0.0159 (14)	0.0156 (14)	−0.0003 (10)	0.0048 (10)	−0.0068 (11)
C1	0.0219 (19)	0.021 (2)	0.018 (2)	0.0006 (15)	0.0063 (14)	−0.0041 (16)
C2	0.0219 (19)	0.0131 (18)	0.019 (2)	−0.0007 (14)	0.0067 (14)	−0.0015 (15)
C3	0.0200 (18)	0.0126 (18)	0.0135 (19)	−0.0019 (13)	0.0069 (13)	0.0019 (14)
C4	0.0190 (18)	0.0121 (18)	0.0139 (19)	0.0019 (13)	0.0034 (13)	0.0016 (14)
C5	0.0183 (18)	0.0131 (17)	0.0106 (18)	−0.0022 (13)	0.0020 (13)	0.0020 (14)
O2	0.0204 (13)	0.0169 (13)	0.0145 (14)	0.0043 (10)	−0.0012 (9)	−0.0007 (11)
C16	0.0218 (19)	0.0155 (19)	0.022 (2)	0.0026 (14)	−0.0017 (14)	0.0030 (16)
C6	0.0232 (19)	0.0093 (17)	0.0111 (18)	−0.0017 (13)	0.0061 (13)	−0.0018 (13)
O3	0.0233 (14)	0.0151 (14)	0.0116 (13)	0.0065 (10)	−0.0012 (9)	−0.0042 (10)
C17	0.0215 (18)	0.0127 (18)	0.0159 (19)	0.0030 (14)	0.0013 (13)	−0.0051 (14)
C7	0.0188 (18)	0.0094 (17)	0.0148 (19)	0.0014 (13)	0.0023 (13)	0.0026 (14)
C8	0.0222 (19)	0.0108 (17)	0.0104 (18)	−0.0027 (13)	0.0047 (13)	0.0012 (14)
C9	0.0247 (19)	0.0114 (17)	0.0105 (18)	−0.0005 (14)	0.0040 (13)	−0.0022 (14)
C10	0.0238 (19)	0.0103 (17)	0.0096 (18)	−0.0011 (13)	0.0004 (13)	−0.0041 (14)
C11	0.026 (2)	0.0077 (17)	0.0091 (18)	0.0035 (13)	−0.0024 (13)	−0.0006 (13)
F1	0.0249 (12)	0.0154 (12)	0.0202 (12)	0.0025 (8)	0.0001 (8)	0.0071 (9)
C12	0.029 (2)	0.0109 (18)	0.0099 (19)	−0.0009 (14)	0.0029 (14)	−0.0023 (14)
C13	0.0215 (19)	0.0170 (19)	0.0169 (19)	0.0021 (14)	0.0009 (14)	−0.0038 (15)
C14	0.030 (2)	0.0125 (18)	0.0148 (19)	0.0064 (14)	−0.0032 (14)	−0.0016 (15)
C15	0.031 (2)	0.0090 (16)	0.0085 (18)	−0.0008 (14)	0.0011 (13)	0.0015 (13)

### Geometric parameters (Å, °)

**Table:**

O1—C1	1.429 (4)	O3—C17	1.432 (4)
O1—C9	1.441 (4)	C17—H17A	0.9800
C1—C2	1.516 (5)	C17—H17B	0.9800
C1—H1A	0.9900	C17—H17C	0.9800
C1—H1B	0.9900	C7—C8	1.391 (5)
C2—C3	1.512 (5)	C7—H7	0.9500
C2—H2A	0.9900	C8—C9	1.525 (5)
C2—H2B	0.9900	C9—C10	1.511 (5)
C3—C8	1.382 (5)	C9—H9	1.0000
C3—C4	1.404 (5)	C10—C11	1.391 (5)
C4—C5	1.384 (5)	C10—C15	1.401 (5)
C4—H4	0.9500	C11—F1	1.360 (4)
C5—O2	1.375 (4)	C11—C12	1.367 (5)
C5—C6	1.408 (5)	C12—C13	1.397 (5)
O2—C16	1.434 (4)	C12—H12	0.9500
C16—H16A	0.9800	C13—C14	1.391 (6)
C16—H16B	0.9800	C13—H13	0.9500
C16—H16C	0.9800	C14—C15	1.379 (5)
C6—O3	1.370 (4)	C14—H14	0.9500
C6—C7	1.391 (5)	C15—H15	0.9500
			
C1—O1—C9	110.9 (3)	H17A—C17—H17B	109.5
O1—C1—C2	110.2 (3)	O3—C17—H17C	109.5
O1—C1—H1A	109.6	H17A—C17—H17C	109.5
C2—C1—H1A	109.6	H17B—C17—H17C	109.5
O1—C1—H1B	109.6	C6—C7—C8	120.9 (3)
C2—C1—H1B	109.6	C6—C7—H7	119.5
H1A—C1—H1B	108.1	C8—C7—H7	119.5
C3—C2—C1	110.6 (3)	C3—C8—C7	120.4 (3)
C3—C2—H2A	109.5	C3—C8—C9	119.5 (3)
C1—C2—H2A	109.5	C7—C8—C9	120.0 (3)
C3—C2—H2B	109.5	O1—C9—C10	106.1 (3)
C1—C2—H2B	109.5	O1—C9—C8	111.6 (3)
H2A—C2—H2B	108.1	C10—C9—C8	116.0 (3)
C8—C3—C4	118.9 (3)	O1—C9—H9	107.6
C8—C3—C2	121.8 (3)	C10—C9—H9	107.6
C4—C3—C2	119.3 (3)	C8—C9—H9	107.6
C5—C4—C3	121.2 (3)	C11—C10—C15	116.5 (3)
C5—C4—H4	119.4	C11—C10—C9	122.5 (3)
C3—C4—H4	119.4	C15—C10—C9	120.9 (3)
O2—C5—C4	124.6 (3)	F1—C11—C12	117.9 (3)
O2—C5—C6	115.8 (3)	F1—C11—C10	118.2 (3)
C4—C5—C6	119.6 (3)	C12—C11—C10	123.8 (3)
C5—O2—C16	115.3 (3)	C11—C12—C13	118.4 (3)
O2—C16—H16A	109.5	C11—C12—H12	120.8
O2—C16—H16B	109.5	C13—C12—H12	120.8
H16A—C16—H16B	109.5	C14—C13—C12	119.9 (3)
O2—C16—H16C	109.5	C14—C13—H13	120.1
H16A—C16—H16C	109.5	C12—C13—H13	120.1
H16B—C16—H16C	109.5	C15—C14—C13	120.1 (3)
O3—C6—C7	125.5 (3)	C15—C14—H14	119.9
O3—C6—C5	115.5 (3)	C13—C14—H14	119.9
C7—C6—C5	119.0 (3)	C14—C15—C10	121.3 (3)
C6—O3—C17	116.5 (3)	C14—C15—H15	119.4
O3—C17—H17A	109.5	C10—C15—H15	119.4
O3—C17—H17B	109.5		
			
C9—O1—C1—C2	69.6 (4)	C6—C7—C8—C9	−176.7 (3)
O1—C1—C2—C3	−47.7 (4)	C1—O1—C9—C10	178.8 (3)
C1—C2—C3—C8	15.5 (5)	C1—O1—C9—C8	−54.0 (4)
C1—C2—C3—C4	−164.0 (3)	C3—C8—C9—O1	20.5 (4)
C8—C3—C4—C5	−0.1 (5)	C7—C8—C9—O1	−163.2 (3)
C2—C3—C4—C5	179.4 (3)	C3—C8—C9—C10	142.2 (3)
C3—C4—C5—O2	179.5 (3)	C7—C8—C9—C10	−41.5 (4)
C3—C4—C5—C6	−0.9 (5)	O1—C9—C10—C11	64.0 (4)
C4—C5—O2—C16	−13.0 (5)	C8—C9—C10—C11	−60.5 (4)
C6—C5—O2—C16	167.4 (3)	O1—C9—C10—C15	−111.9 (3)
O2—C5—C6—O3	−0.4 (4)	C8—C9—C10—C15	123.6 (3)
C4—C5—C6—O3	−179.9 (3)	C15—C10—C11—F1	−178.8 (3)
O2—C5—C6—C7	−179.2 (3)	C9—C10—C11—F1	5.1 (5)
C4—C5—C6—C7	1.2 (5)	C15—C10—C11—C12	1.1 (5)
C7—C6—O3—C17	−4.7 (5)	C9—C10—C11—C12	−174.9 (3)
C5—C6—O3—C17	176.6 (3)	F1—C11—C12—C13	179.9 (3)
O3—C6—C7—C8	−179.3 (3)	C10—C11—C12—C13	0.0 (5)
C5—C6—C7—C8	−0.6 (5)	C11—C12—C13—C14	−1.0 (5)
C4—C3—C8—C7	0.8 (5)	C12—C13—C14—C15	0.8 (5)
C2—C3—C8—C7	−178.7 (3)	C13—C14—C15—C10	0.3 (5)
C4—C3—C8—C9	177.1 (3)	C11—C10—C15—C14	−1.3 (5)
C2—C3—C8—C9	−2.4 (5)	C9—C10—C15—C14	174.8 (3)
C6—C7—C8—C3	−0.5 (5)		

### Hydrogen-bond geometry (Å, °)

**Table:**

*D*—H···*A*	*D*—H	H···*A*	*D*···*A*	*D*—H···*A*
C1—H1B···O2^i^	0.99	2.59	3.360 (5)	134
C7—H7···F1^ii^	0.95	2.45	3.360 (4)	160
C17—H17B···O1^iii^	0.98	2.49	3.430 (4)	160
C17—H17A···Cg^ii^	0.98	2.70	3.557 (3)	146

Symmetry codes: (i) *x*, −*y*+3/2, *z*−1/2; (ii) *x*, *y*+1, *z*; (iii) *x*, −*y*+5/2, *z*+1/2.
